# Effects of *Bacillus coagulans* on growth performance and intestinal mucosal barrier functions of juvenile channel catfish (*Ictalurus punctatus*)

**DOI:** 10.3389/fimmu.2026.1909579

**Published:** 2026-09-03

**Authors:** Huige Ren, Zihe Guo, Xiao Peng, Ye Qian, Chanxia Qin, Jingyi Du, Chengrui Huang, Sikai Huang, Yonggang Mo, Weihao Ou

**Affiliations:** Key Laboratory of Aquatic Healthy Breeding and Nutrition Regulation of Guangxi Universities, College of Animal Science and Technology, Guangxi University, Nanning, Guangxi, China

**Keywords:** *Bacillus coagulans*, growth performance, *Ictalurus punctatus*, intestinal metabolism, intestinal mucosal barrier

## Abstract

To investigate the effects of *Bacillus coagulans* on growth performance and intestinal mucosal barrier functions of juvenile channel catfish (*Ictalurus punctatus*), this study formulated two experimental diets: a control diet (CN group, without *B. coagulans*) and a *B. coagulans*-supplemented diet (BC group, containing 1×10^8^ CFU/g *B. coagulans*). Each diet was assigned to three replicate tanks for an 8-week feeding trial. Compared with the CN group, the BC group exhibited a significantly lower feed conversion ratio (*P* < 0.05). The BC group exhibited significantly increased activities of intestinal trypsin, α-amylase, and glutathione S-transferase (*P* < 0.05), alongside significantly reduced levels of malondialdehyde and superoxide anion (*P* < 0.05). Moreover, the BC group showed significantly upregulated gene expression of intestinal tight junction proteins (*zonula occludens-2*, *claudin-18*, *claudin-g*, *junctional adhesion molecule-2b*, and *junctional adhesion molecule-3b*; *P* < 0.05) and the anti-inflammatory factor (*transforming growth factor-β*; *P* < 0.05). Conversely, the gene expression levels of pro-inflammatory factors (*interleukin-1β*, *tumor necrosis factor-α*, *interleukin-6*, and *interferon-γ1*) were significantly downregulated (*P* < 0.05). Additionally, genes associated with the intestinal chemical barrier (*mucin-4*, *lysozyme-c*, *lysozyme-g*, *NK-lysin type 1*, and *β-defensin*) were significantly upregulated (*P* < 0.05). Intestinal microbiota analysis revealed increased relative abundance of beneficial bacteria (e.g., Firmicutes, Bacilli, Erysipelotrichales, and *Turicibacter*) in the BC group (*P* < 0.05). Non-targeted metabolomics further indicated significantly elevated levels of beneficial metabolites, including soyasaponin I and taurine in the BC group (*P* < 0.05). In summary, dietary *B. coagulans* improved feed utilization, enhanced intestinal digestive and antioxidant capacity, strengthened mucosal barrier functions, optimized intestinal metabolism, and promoted intestinal health.

## 1 Introduction

Intensive aquaculture plays an increasingly vital role in meeting the growing global demand for high-quality animal protein (1, 2). However, high-density farming environments are prone to inducing intestinal dysfunction and disrupting immune homeostasis in aquatic animals, thereby significantly increasing the risk of disease outbreaks (3–5). The intestine serves not only as a key organ for nutrient absorption but also as a crucial immune defense interface, where the stability of the mucosal barrier directly determines the host’s resistance to pathogenic invasion (6–8). In aquaculture practice, antibiotics are widely used to mitigate health risks caused by pathogen infections (9); however, the long-term and improper use of antibiotics may lead to multiple adverse effects, including the induction of antimicrobial resistance in bacteria, negative impacts on host health, and issues related to antibiotic residues (9–11). Therefore, the development of environmentally friendly alternatives targeting intestinal health and immune homeostasis has become a major focus in aquaculture nutrition and immunology research.

Probiotics have garnered extensive attention as functional feed additives due to their ability to modulate gut microbiota, improve nutrient utilization, and enhance host immunity (3, 5, 9, 10, 12). Among these, *Bacillus coagulans*, a functional strain combining spore stability with the metabolic characteristics of lactic acid bacteria (13), has increasingly gained attention in aquaculture. Although this strain lacks long-term colonization capacity in the intestine, its metabolites such as lactic acid, antimicrobial substances, and various digestive enzymes can improve the host’s physiological and immune status by modulating the intestinal microbiota, enhancing antioxidant defense, and activating immune-related signaling pathways (14, 15). Studies in Nile tilapia (*Oreochromis niloticus*) (16), common carp (*Cyprinus carpio*) (17), crucian carp (*Carassius auratus*) (13), giant freshwater prawn (*Macrobrachium rosenbergii*) (18), and other aquatic species have demonstrated that dietary supplementation with *B. coagulans* can significantly promote growth performance and improve intestinal health, with an optimal inclusion level of approximately 1 × 10^8^ CFU g^-1^ in aquafeeds (17, 19–21). However, current research has mainly focused on its effects on growth and general immune parameters, while its regulation of intestinal mucosal barrier function and the underlying microbiota-mediated mechanisms remains poorly understood.

The intestinal mucosal barrier serves as a critical line of defense in aquatic animals, protecting against pathogen invasion and maintaining immune homeostasis (22, 23). It is composed of the physical barrier formed by tight junction proteins, the chemical barrier consisting of mucus and antimicrobial substances, the immune barrier mediated by immune cells and cytokines, and the biological barrier established by commensal microbiota (22–26). Disruption of this barrier is closely associated with intestinal inflammation and disease outbreaks (6, 7). Probiotics participate in mucosal barrier repair and immune regulation by modulating the microbiota structure and associated metabolites, which is considered an important mechanism for their protective effects (27, 28). However, research on the related mechanisms of *B. coagulans* remains very limited.

Channel catfish (*Ictalurus punctatus*) is one of the important freshwater economic fish species in China (29, 30). Under intensive farming conditions, channel catfish are highly susceptible to intestinal diseases, which are frequently associated with impaired mucosal barrier function (31–33). However, to date, studies investigating probiotic-mediated regulation of intestinal barrier function in channel catfish are scarce, and neither the dietary application of *B. coagulans* nor its mechanisms of action have been investigated in channel catfish. Therefore, this study aimed to systematically evaluate the effects of dietary supplementation with *B. coagulans* on the growth performance and intestinal mucosal barrier function of juvenile channel catfish, thereby providing a theoretical basis for its application in aquaculture.

## 2 Materials and methods

### 2.1 Experimental diets

The basal diet (Model No. 661; crude protein 37%, crude fiber 8%, crude ash 15%, and crude lipid 5%) was purchased from Zhanjiang Haida Feed Co., Ltd. (Zhanjiang, China). *B. coagulans* (CGMCC NO.13044) spore powder (1 × 10^10^ CFU g^−1^) was acquired from Qingdao GBW Group Co., Ltd. (Qingdao, China). For the BC group diet, the proper amount of *B. coagulans* spore powder was suspended in 60 mL of sterile ultrapure water, thoroughly vortexed to ensure homogeneity, and evenly sprayed onto 500 g of the basal diet and mixed manually to achieve a final concentration of 1 × 10^8^ CFU g^−1^. The coated feed was dried at 50 °C for 2 h and stored in sealed plastic bags at 4 °C in the dark until use. The CN group diet was prepared using the same procedure, including spraying with an equal volume of sterile ultrapure water and drying at 50 °C for 2 h, without *B. coagulans* supplementation. Fresh diets for both groups were prepared weekly to ensure probiotic viability. For each batch, three aliquots of the coated feed were randomly sampled after mixing and drying, and CFU enumeration by plate counting was performed to verify the target concentration and coating homogeneity.

### 2.2 Feeding trial

Juvenile channel catfish used in this experiment were obtained from an aquaculture farm in Nanning, Guangxi, China. Fish were acclimated to the rearing environment by feeding with the basal diet for 2 weeks. After 24 h of fasting, fish (13.50 ± 0.21 g) were randomly divided into the control group (without *B. coagulans*; CN group) and the treatment group (with *B. coagulans*; BC group). Each group consisted of three 400 L plastic tanks (30 fish per tank), with continuous aeration. Fish were fed twice daily (08:00 and 16:00) to apparent satiation. The feeding trial lasted 8 weeks (under a natural photoperiod). One-third of the water in each tank was replaced daily with dechlorinated and aerated tap water. Uneaten feed was collected by siphoning approximately 30 min after each feeding, dried, and weighed to determine actual daily feed intake per tank. Feces were promptly removed to prevent water quality deterioration (temperature 26–30 °C; dissolved oxygen > 7.0 mg L^−1^; pH 7.5–8.2; ammonia nitrogen ≤ 0.2 mg L^−1^; nitrite < 0.1 mg L^−1^).

### 2.3 Sample collection

At the end of the feeding trial, fish were fasted for 24 h, anesthetized with an appropriate dose of eugenol, and then weighed and counted. All hindgut samples (without intestinal contents) were collected with sterilized scissors and forceps under aseptic conditions. Hindgut samples from 2 randomly selected fish within each tank were pooled. Each group ultimately included 3 pooled samples for intestinal enzyme activity analysis, 3 pooled samples for intestinal gene expression analysis, 3 pooled samples for intestinal microbiota analysis, and 6 pooled samples for intestinal metabolite analysis. Each pooled sample was placed in sterile, DNase/RNase-free 2 mL centrifuge tubes, then immediately flash-frozen in liquid nitrogen, and finally stored at −80 °C.

### 2.4 Growth performance

The growth performance parameters were calculated using the following formulas:

Weight gain rate (WGR, %) = 100 × (FBW (g) − IBW (g))/IBW (g);
Specific growth rate (SGR, %/day) =100 × [ln (FBW (g)) − ln (IBW (g))]/days;
Feed conversion ratio (FCR) = feed intake (g)/(FBW (g) − IBW (g));
Survival rate (SR, %) = 100 × Nt/Ng.

Notes: Nt is final number of fish; Ng is initial number of fish; IBW is initial body weight; FBW is final body weight.

### 2.5 Intestinal digestive and antioxidant enzyme activities

Hindgut tissue samples were weighed (approximately 100 mg), homogenized in 0.9 mL of ice-cold phosphate-buffered saline (PBS, pH 7.4), and centrifuged at 3,000 *g* for 10 min at 4 °C. The supernatant was collected for the measurement of the following parameters using commercial assay kits. The following intestinal parameters were measured using assay kits from Nanjing Jiancheng Bioengineering Institute (Nanjing, China): Total protein content (Model Number A045-4-2), α-amylase activity (Model Number C016-1-1), lipase activity (Model Number A054-1-1), trypsin activity (Model Number A080-2-2), catalase (CAT) activity (Model Number A007-1-1), total superoxide dismutase (T-SOD) activity (Model Number A001-3-2), and malondialdehyde (MDA) content (Model Number A003-1-2). In addition, intestinal glutathione S-transferase (GST) activity (Model Number BC0355) and superoxide anion (O_2_^−^) content (Model Number BC1295) were determined using assay kits from Beijing Solarbio Science & Technology Co., Ltd. (Beijing, China). Enzyme activities were normalized to total protein concentration determined by the Bradford method. All experimental procedures were performed in accordance with the manufacturers’ instructions.

### 2.6 Gene expression related to intestinal mucosal barrier function

Genes associated with the intestinal mechanical barrier, immune barrier, and chemical barrier were analyzed. Total RNA was extracted from intestinal samples using the Trizol method. The extracted RNA was electrophoresed on a 1.2% (w/v) denaturing agarose gel to assess the integrity followed by determination of concentration with UV-Vis Spectrophotometer Q5000 (Quawell Technology Inc., San Jose, CA, USA). The detection results demonstrated that the quality and quantity of the extracted RNA were satisfactory. Subsequently, the extracted RNA was reverse-transcribed into cDNA using a qPCR-specific reverse transcription kit (Model Number RR047A; Takara Biomedical Technology (Beijing) Co., Ltd., Beijing, China). Real-time PCR was performed using the CFX384 Touch™ Real-Time PCR Detection System (Bio-Rad Laboratories, Inc., Hercules, CA, USA). Real-time PCR reaction system was as follows: cDNA 1 μL, forward primer (10 μM) 0.5 μL, Reverse primer (10 μM) 0.5 μL, 2×RealStar Fast SYBR qPCR mix 10 μL (Model Number A301-10; Genstar, Beijing, China), and ddH_2_O 8 μL. Real-time PCR thermal cycling conditions were as follows: 95 °C 2 min, followed by 40 cycles of “95 °C (15 s), 60 °C (30 s), and 72 °C (30 s)”. Melt curve analysis was performed after amplification to verify primer specificity, and a single peak was observed for all primer pairs, indicating specific amplification. The amplification efficiency of each primer pair was determined using a standard curve generated from twofold serial dilutions of pooled cDNA; all efficiencies ranged between 90% and 110%. The 18S rRNA gene was used as the reference gene. Gene expression levels were analyzed using the 2^−ΔΔCT^ method. The relevant primer sequences are listed in **Table 1**.

**Table 1:** Primer sequences for real-time PCR.

Gene	Primer	Sequence 5’–3’	GenBank no.
*occludin*	F	TCATTTGACGGGGACTTGTGT	XM_017467503.3
	R	CTGGGCGTGTGAGTGAGAAAC	
*zo-2*	F	GGACGCGGAAGTGTGACAT	XM_017488927.3
	R	TCGGATTGTCTTTTCCGCCA	
*claudin-31*	F	CCCATTTTGGCGCGTATCAG	NM_001329250.1
	R	CCCCAACTGTGATAGGCAGG	
*claudin-18*	F	AAACCTTGAGGTCCCTCCCT	NM_001329288.1
	R	GCTCCAGTTGTTCATTGCGG	
*claudin-g*	F	TATGGTGGTTCTGTCTGGTGC	NM_001329251.1
	R	ACCAAGCATCTGTAGCCCT	
*Jam-2b*	F	CGGTCTCCCTCAATGTGCTT	XM_017469796.3
	R	CCACCACAGAGCCACTCATT	
*Jam-3b*	F	CAGAGCAAAGATTCGCGAGC	XM_047161997.2
	R	GCGGTACGATGCAGTGTCTA	
*il-1β*	F	CGGAGGCTCATTTTATGTTTATCT	NM_001200220.1
	R	ACAGAGCAGTCACATATCTCACAA	
*tnf-α*	F	TATGTAAAGCCGAGGGCGAC	NM_001200172.1
	R	GAGTCAGACCAGCGAGACAC	
*il-6*	F	CGACCTCCTTACCTGATGCTT	OM641812.1
	R	CTTCATGTAATTGATCGCCCTG	
*il-17a*	F	TGGTTGCTCAGGCTGCTCCTT	XM_017455432.3
	R	ACGCCAGCTTGATGTCATGTTCC	
*ifn-γ1*	F	ATGTGCGGACTTGTCATGGT	DQ124249.1
	R	TCACAAGTGCAGGTGTCCTC	
*tgf-β*	F	CGTTTCCGCTTCAAGATGGC	XM_017482335.3
	R	GTGCATTTGGTTGCTTTTGCC	
*mucin-4*	F	CCAGAATATGTACAGGAGCGTT	KY490705.1
	R	TGCAGGTGGCAGAAATAGGAC	
*leg*	F	AGCATCATTCCAATAAACCCCC	NM_001200929.1
	R	ACAAGAAGCAAAACATCGCTGA	
*lysozyme-c*	F	GATGGATCAACGGACTATG	NM_001200789.1
	R	CTGTCTCACTATGGTCTTG	
*lysozyme-g*	F	CATCGGAAATAACAGCCAAG	NM_001200819.2
	R	TCTCTGGATATAATGCCTGC	
*NK-lysin type 1*	F	CTGAGACACAGCTACTCCCTG	NM_001200208.1
	R	TTGCAGAGACCTCGAAGGAAT	
*β-defensin*	F	TCCTGCTGGTCATCTTAGCAC	KX211992.1
	R	ACACGCAGCATAAAGATCCCT	
*18S*	F	GCAATTATTCCCCATGAACGAGG	XR_008395261.1
	R	GGACCTCACTAAACCATCCGA	

F, forward primer; R, reverse primer. Abbreviations are defined as follows: *zo-2, Zonula Occludens-2; jam-2b, junctional adhesion molecule-2b; jam-3b, junctional adhesion molecule-3b; il-1β, Interleukin-1β; tnf-α, Tumor Necrosis Factor-α; il-6, Interleukin-6; il-17a, Interleukin-17a; ifn-γ1, Interferon Gamma 1; tgf-β, Transforming Growth Factor-β; leg, beta-galactoside-binding lectin; 18S, 18S ribosomal RNA*. All primer sequences were verified by NCBI Primer-BLAST.

### 2.7 Intestinal microbiota analysis

The genomic DNA of the gut microbiota from intestinal samples was extracted using the CTAB method. The V4 region of the 16S rRNA gene was amplified. All PCR mixtures contained 15 µL of Phusion^®^ High-Fidelity PCR Master Mix (New England Biolabs, Inc., Ipswich, MA, USA), 0.2 µM primers, and 10 ng of genomic DNA template. The initial denaturation was performed at 98 °C for 1 min, followed by 30 cycles of “98 °C (10 s), 50 °C (30 s), and 72 °C (30 s)”, with a final extension at 72 °C for 5 min. The PCR products were then purified using magnetic beads, pooled in equal amounts based on their concentrations, thoroughly mixed, and detected to recover the target bands. Finally, a library was constructed, and the quality of the library was assessed using Qubit and qPCR. After passing the quality check, the library was sequenced on the NovaSeq 6000 platform using PE250 mode, yielding an average of approximately 100,000 raw PE reads per sample. The raw data of 16S rRNA gene amplicon sequencing had been uploaded to the Sequence Read Archive (SRA) with the accession number PRJNA1450605.

The sequencing data were demultiplexed based on barcode sequences and PCR amplification primer sequences. After removing the barcode and primer sequences, FLASH (34) was used to assemble the reads for each sample, generating Raw Tags data. Cutadapt software was then employed to match and trim the reverse primer sequences to prevent interference with subsequent analyses. The Raw Tags were rigorously filtered using fastp software (Version 0.23.1) to obtain high-quality Clean Tags. The resulting tag sequences were compared against the Silva database (https://www.arb-silva.de/for16S/18S (accessed on 2 September 2024)) to detect and remove chimeric sequences, yielding the final effective data (Effective Tags) (35). The Effective Tags were further denoised to obtain Amplicon Sequence Variants (ASVs) and a feature table (36). Taxonomic annotation was performed using QIIME2 software with the Silva 138.1 database. The data from each sample were normalized to the sample with the lowest sequencing depth. Venn diagrams were generated using the VennDiagram function in R. Species abundance statistics were calculated for the top ten species at different taxonomic levels (phylum and genus), and relative abundance distribution histograms were plotted using the SVG function in Perl. Principal co-ordinate analysis (PCoA) was performed and visualized using the ade4 and ggplot2 packages in R (V4.0.3). α-diversity indices, including Chao1, Shannon, Simpson, Pielou_e, and Good’s coverage, were calculated using QIIME2, and rarefaction curves were generated. Differential microbial communities between groups were analyzed using LDA Effect Size (LEfSe) (37). Functional prediction was conducted using PICRUSt.

### 2.8 Intestinal LC-MS untargeted metabolomics analysis

After precisely weighing 50 ± 5 mg of each intestinal sample, it was placed into a 2 mL centrifuge tube along with a 6 mm grinding bead and 400 µL extraction solution (methanol–water = 4:1 (v:v)), containing four internal standards (L-2-chlorophenylalanine (0.02 mg/mL), etc.). The sample was homogenized using a cryogenic tissue homogenizer for 6 min (−10 °C, 50 Hz), followed by low-temperature ultrasonic extraction for 30 min (5 °C, 40 KHz). The sample was then incubated at −20 °C for 30 min and centrifuged for 15 min (13,000× g, 4 °C). The supernatant was collected for analysis using an LC-MS/MS system UHPLC-Triple TOF 6600 (SCIEX, Foster City, CA, USA). Quality control (QC) samples were prepared by mixing equal volumes of extraction solution from all samples. A QC sample was inserted after every 5–15 analytical samples to assess the stability of the detection process. The raw data were imported into the metabolomics processing software ProgenesisQI v3.0 (Waters Corporation, Milford, MA, USA) for baseline filtering, peak identification, integration, retention time correction, and peak alignment. This resulted in a data matrix containing information on retention time, m/z ratio, and peak intensity. Feature peak library identification was performed using this software by matching MS and MS/MS spectral information with metabolomics databases. The MS mass error was set to less than 10 ppm, and metabolites were identified based on secondary mass spectrometry matching scores. Key databases included http://www.hmdb.ca/ (accessed on 22 October 2024) and https://metlin.scripps.edu/ (accessed on 22 October 2024), as well as a self-built database. Principal component analysis (PCA) and multivariate statistical analysis of differential metabolites were conducted using ropls (R packages, Version 1.6.2). KEGG compound classification and functional pathway analysis were performed using the KEGG database (kegg_v20230830, Release 2017-05-01). KEGG pathway enrichment, heatmaps, clustering analysis, and correlation analysis were implemented using scipy (Python, Version 1.0.0). Variable importance in projection (VIP) analysis was performed using a combination of ropls (R packages, Version 1.6.2) and scipy (Python, Version 1.0.0). Sankey diagrams were generated using the Majorbio Cloud Platform (https://analysis.majorbio.com/) with default parameters for visualization of metabolite–pathway associations.

### 2.9 Statistical analysis

All data were presented as mean ± SEM. Student’s t-tests were performed for statistical analysis after confirming data normality and homogeneity of variance. GraphPad Prism 9.0 software (GraphPad, San Diego, CA, USA) was used for plotting. ns indicates *P* > 0.05, * indicates *P* < 0.05, ** indicates *P* < 0.01, *** indicates *P* < 0.001.

## 3 Results

### 3.1 Growth performance

**Figure 1:**
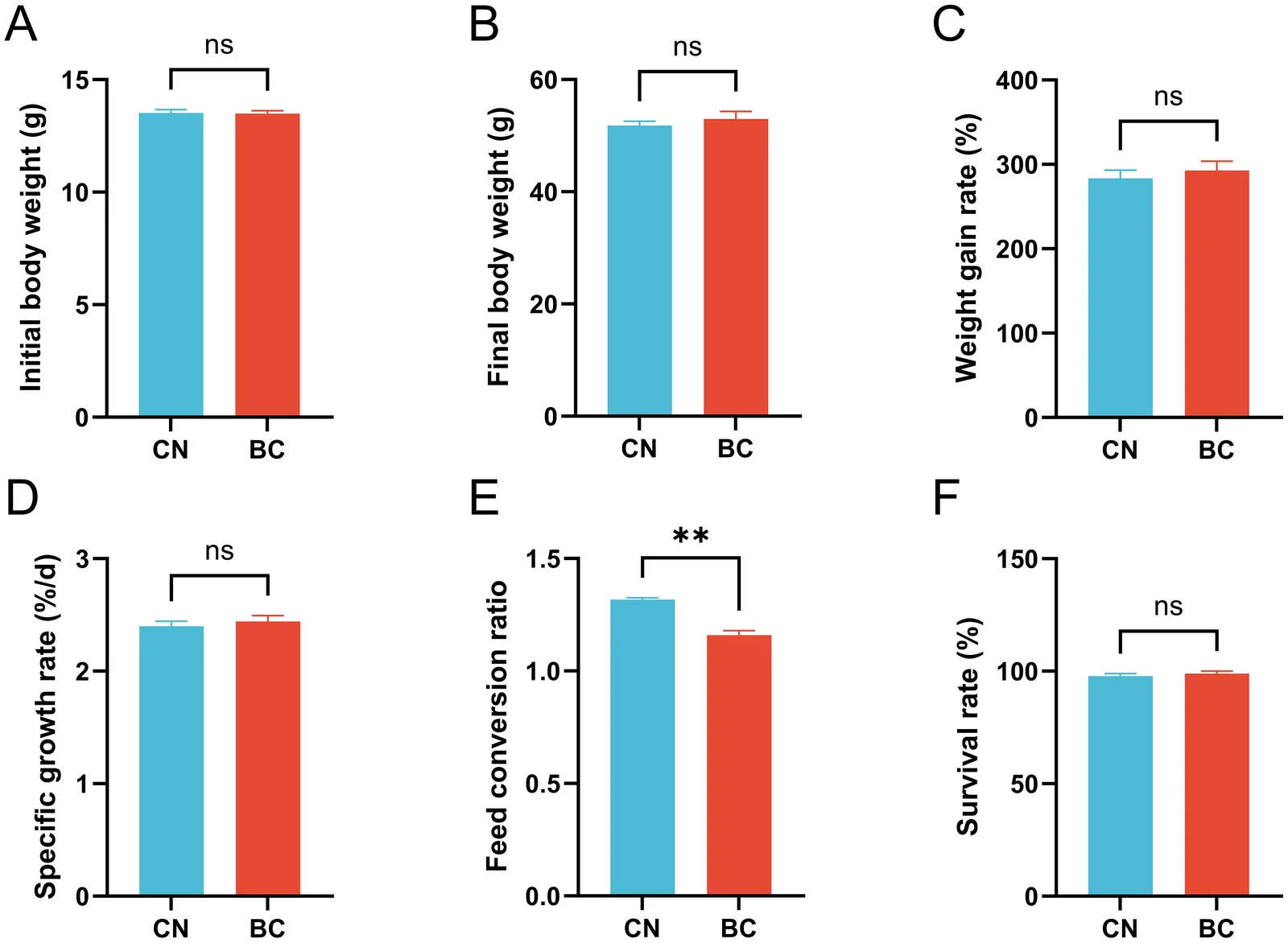
Effects of *Bacillus coagulans* on growth performance of juvenile channel catfish. **(A)** Initial body weight; **(B)** Final body weight; **(C)** Weight gain rate; **(D)** Specific growth rate; **(E)** Feed conversion ratio; **(F)** Survival rate; ns indicates *P* > 0.05, * indicates *P* < 0.05, ** indicates *P* < 0.01, and *** indicates *P* < 0.001.

To evaluate the effects of dietary supplementation with *Bacillus coagulans* on the growth performance of juvenile channel catfish, this study measured IBW, FBW, WGR, SGR, FCR, and SR. The results showed that compared with the CN group, the BC group showed no significant differences in IBW, FBW, WGR, SGR, and SR (*P* > 0.05) (**Figures 1A–D, F**). By contrast, the FCR in the BC group was significantly lower than that in the CN group (*P* < 0.01) (**Figure 1E**).

### 3.2 Intestinal digestive and antioxidant enzyme activities

**Figure 2:**
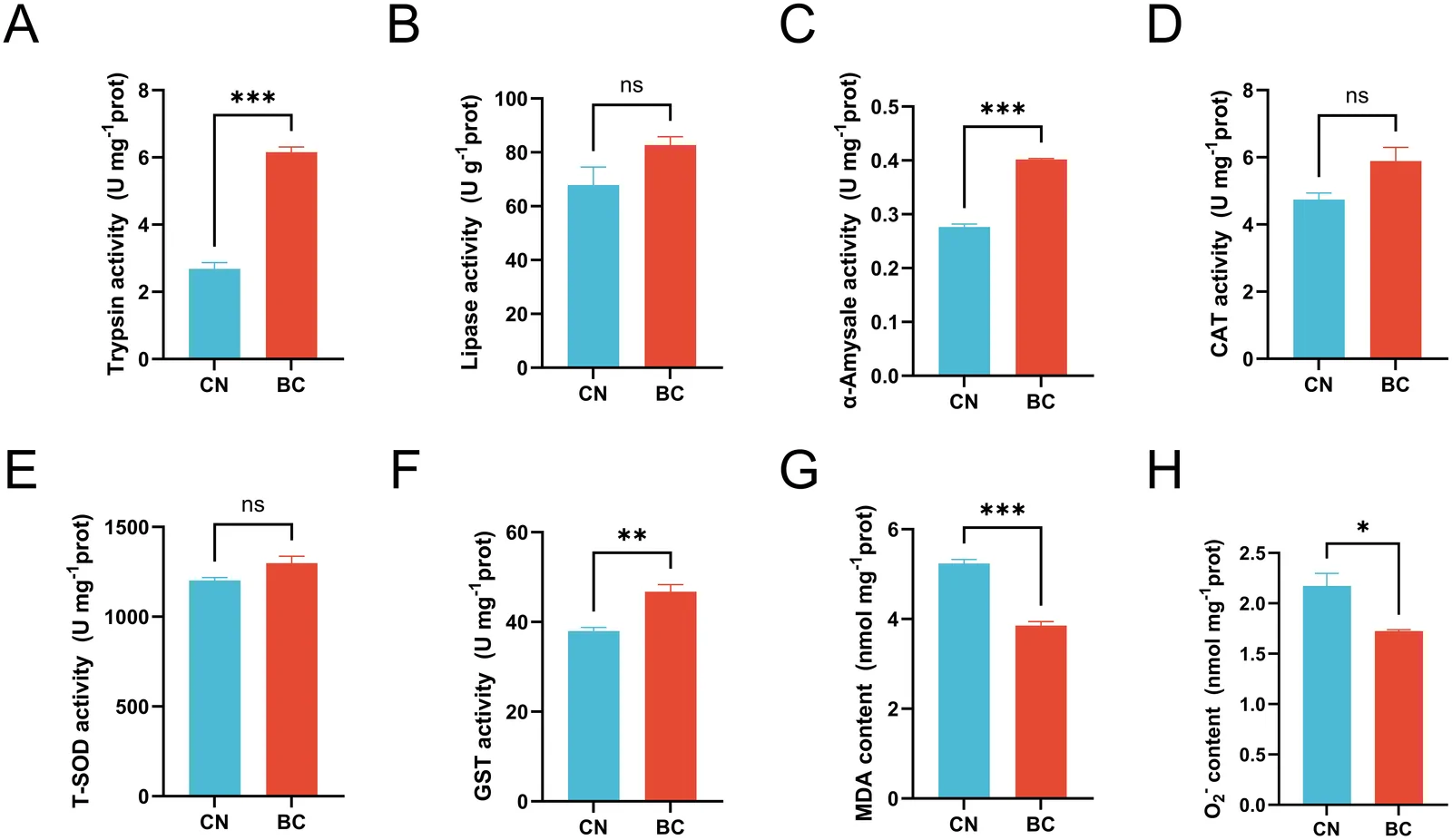
Effects of *Bacillus coagulans* on intestinal digestive and antioxidant enzyme activities of juvenile channel catfish. **(A)** Trypsin activity; **(B)** Lipase activity; **(C)** α-Amylase activity; **(D)** Catalase (CAT) activity; **(E)** Total superoxide dismutase (T-SOD) activity; **(F)** Glutathione S-transferase (GST) activity; **(G)** Malondialdehyde (MDA) content; **(H)** Superoxide anion (O_2_^−^) content; ns indicates *P* > 0.05, * indicates *P* < 0.05, ** indicates *P* < 0.01, and *** indicates *P* < 0.001.

To investigate the effects of dietary supplementation with *Bacillus coagulans* on intestinal digestive enzymes, antioxidant enzyme activities, and related biochemical indicators in juvenile channel catfish, this study measured the activities of intestinal trypsin, lipase, α-amylase, CAT, T-SOD, GST, and the contents of MDA and O_2_^−^. The results showed that the activities of trypsin (*P* < 0.001), α-amylase (*P* < 0.001), and GST (*P* < 0.01) in the BC group were significantly higher than those in the CN group (**Figures 2A, C, F**). Meanwhile, MDA content (*P* < 0.001) and O_2_^−^ content (*P* < 0.05) in the BC group were significantly lower than those in the CN group (**Figures 2G, H**). However, no significant differences were observed in the activities of lipase, CAT, or T-SOD between the two groups (*P* > 0.05) (**Figures 2B, D, E**).

### 3.3 Gene expression related to intestinal mucosal barrier function

**Figure 3:**
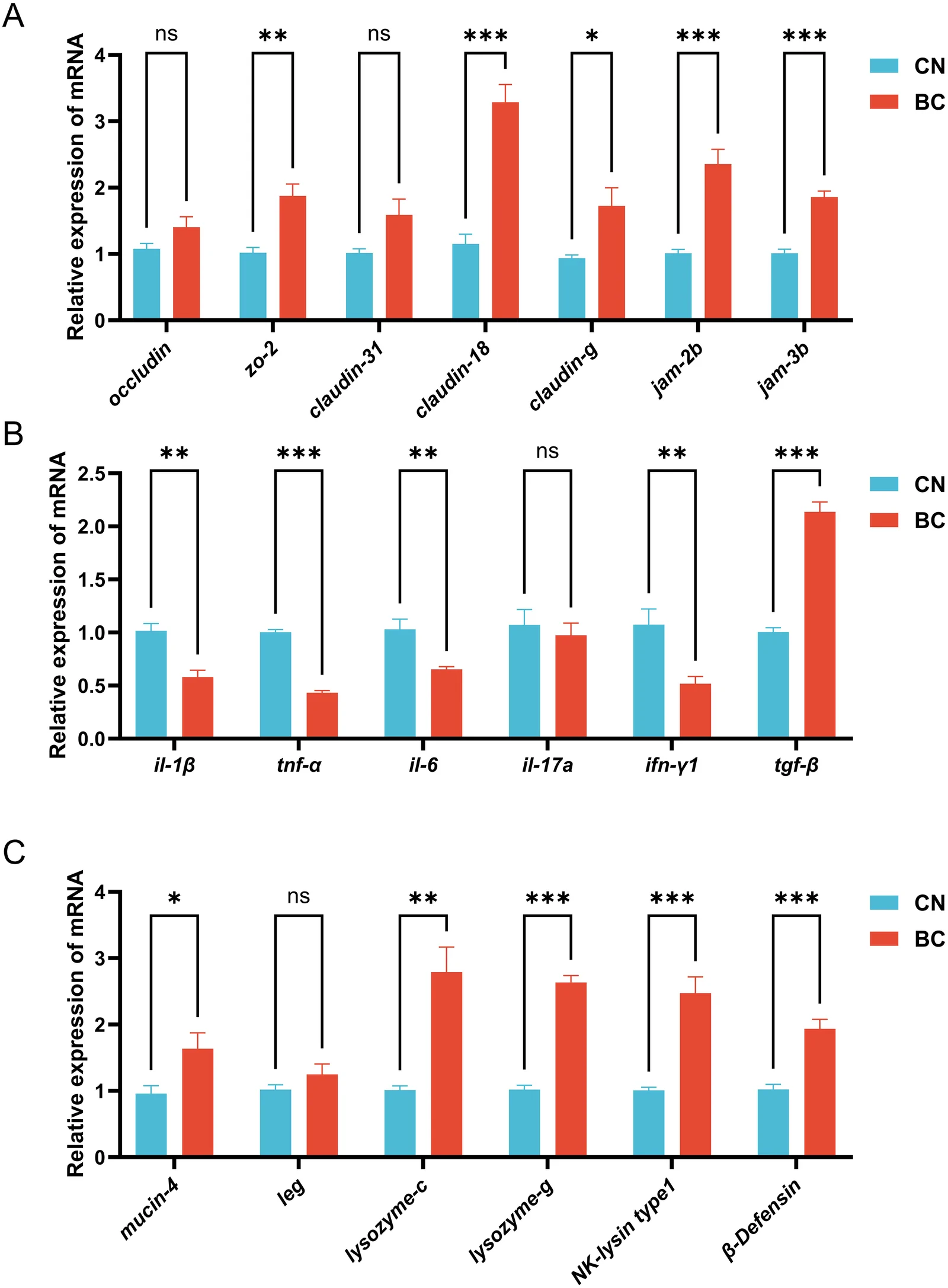
Effects of *Bacillus coagulans* on gene expression related to intestinal mucosal barrier function of juvenile channel catfish. **(A)** Tight junction-related gene expression; **(B)** Inflammatory cytokine gene expression; **(C)** Chemical barrier-related gene expression; ns indicates *P* > 0.05, * indicates *P* < 0.05, ** indicates *P* < 0.01, and *** indicates *P* < 0.001.

To further elucidate the effects of dietary supplementation with *Bacillus coagulans* on the intestinal mucosal barrier function of juvenile channel catfish, this study measured the expression levels of tight junction proteins in the mechanical barrier, inflammatory cytokines in the immune barrier, and chemical barrier-related genes. The results showed that compared with the CN group, in terms of the mechanical barrier, the BC group exhibited significantly upregulated gene expression of *zo-2* (*P* < 0.01), *claudin-18* (*P* < 0.001), *claudin-g* (*P* < 0.05), *jam-2b* (*P* < 0.001), and *jam-3b* (*P* < 0.001), whereas no significant differences were observed in the gene expression of *occludin* and *claudin-31* (*P *> 0.05) (**Figure 3A**). Regarding the immune barrier, the BC group showed significantly downregulated gene expression of the pro-inflammatory cytokines *il-1β* (*P* < 0.01), *tnf-α* (*P* < 0.001), *il-6* (*P* < 0.01), and *ifn-γ1* (*P* < 0.01), significantly upregulated gene expression of the anti-inflammatory cytokine *tgf-β* (*P* < 0.001), and no significant difference in the gene expression of *il-17a* (*P* > 0.05) (**Figure 3B**). For the chemical barrier, the BC group demonstrated significantly upregulated gene expression of *mucin-4* (*P* < 0.05), *lysozyme-c* (*P* < 0.01), *lysozyme-g* (*P* < 0.001), *NK-lysin type 1* (*P* < 0.001), and *β-defensin* (*P* < 0.001), while no significant difference was found in the gene expression of *leg* (*P* > 0.05) (**Figure 3C**).

### 3.4 Intestinal microbiota

**Figure 4:**
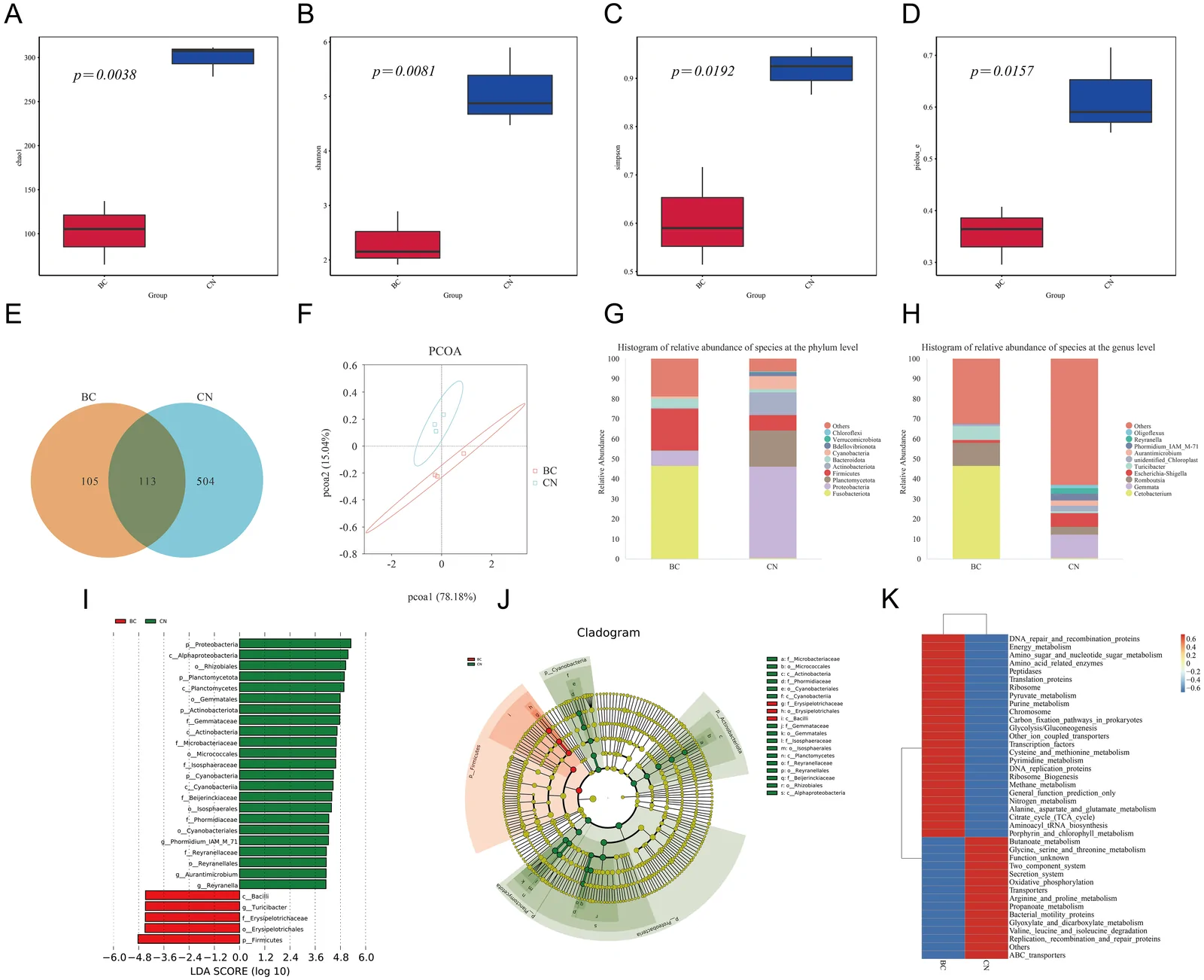
Effects of *Bacillus coagulans* on intestinal microbiota of juvenile channel catfish. **(A)** Chao1 index; **(B)** Shannon index; **(C)** Simpson index; **(D)** Pielou_e index; **(E)** Venn diagram; **(F)** Principal coordinates analysis (PCoA) plot; **(G)** Top ten bacterial phyla ranked by relative abundance; **(H)** Top ten bacterial genera ranked by relative abundance; **(I)** LDA effect size (LEfSe) analysis (LDA score > 4); **(J)** LEfSe cladogram; **(K)** Heatmap of predicted microbial functional profiles based on PICRUSt (representative KEGG level 3 categories).

α-diversity reflects information such as the richness and evenness of microbial communities, thereby indicating the stability of the intestinal microecosystem across different groups. Rarefaction curves for Shannon index and Simpson index approached a plateau, indicating sufficient sequencing depth (**Supplementary Figures 1**, **2**). In this study, α-diversity between the two groups was assessed using the Chao1, Shannon, Simpson, Pielou_e, and Good’s coverage indices. As shown in **Figures 4A–D** and **Supplementary Figure 3**, compared with the CN group, the Chao1, Shannon, Simpson, and Pielou_e indices were significantly reduced in the BC group (*P* < 0.05), whereas Good’s coverage did not differ significantly between the two groups (*P* > 0.05). To assess differences in intestinal microbial composition between the CN and BC groups, the number of ASVs and β-diversity were analyzed. The Venn diagram showed that the CN and BC groups shared 113 ASVs, while the CN and BC groups had 504 and 105 unique ASVs, respectively (**Figure 4E**). In addition, β-diversity analysis based on PCoA revealed that samples from the CN and BC groups formed clearly separated clusters in the ordination plot (**Figure 4F**). These results indicated that the composition and structure of the intestinal microbiota differed markedly between the two groups.

To characterize the dominant composition of the intestinal microbiota across different groups, this study analyzed the relative abundances of microbial taxa at both the phylum and genus levels. The results revealed that at the phylum level, Chloroflexi, Verrucomicrobiota, Bdellovibrionota, Cyanobacteria, Bacteroidota, Actinobacteriota, Firmicutes, Planctomycetota, Proteobacteria, and Fusobacteriota were the top ten dominant phyla in terms of relative abundance (**Figure 4G**). At the genus level, the top ten dominant genera were *Oligoflexus*, *Reyranella*, *Phormidium*_IAM M-71, *Aurantimicrobium*, unidentified_Chloroplast, *Turicibacter*, *Escherichia-Shigella*, *Romboutsia*, *Gemmata*, and *Cetobacterium* (**Figure 4H**).

To further identify the biomarker taxa of the intestinal microbiota between the CN and BC groups, this study employed LEfSe analysis (**Figure 4I**). The results showed that the CN group was enriched in microbial taxa represented by Proteobacteria, Planctomycetota, Actinobacteriota, and Cyanobacteria across multiple taxonomic levels. These taxa were mainly distributed among the orders Rhizobiales, Gemmatales, Micrococcales, Isosphaerales, Cyanobacteriales, and Reyranellales, and at the genus level were represented by *Phormidium* IAM M-71, *Aurantimicrobium*, and *Reyranella*. In contrast, the biomarker taxa in the BC group were mainly concentrated within the phylum Firmicutes and were represented at the class, order, and family levels by Bacilli, Erysipelotrichales, and Erysipelotrichaceae, respectively, with *Turicibacter* identified as the representative genus (**Figure 4I**). The LEfSe cladogram further indicated that the biomarker taxa of the BC group were primarily affiliated with the phylum Firmicutes, whereas those of the CN group were mainly distributed among the phyla Cyanobacteria, Actinobacteriota, Proteobacteria, and Planctomycetota (**Figure 4J**).

The PICRUSt-based functional prediction results (**Figure 4K**) showed that the abundances of multiple functional pathways differed between the BC and CN groups. For example, the BC group exhibited significantly higher abundances of pathways related to DNA repair and recombination proteins, Energy metabolism, Amino sugar and nucleotide sugar metabolism, Glycolysis/Gluconeogenesis, Pyruvate metabolism, Citrate cycle (TCA cycle), Purine metabolism, Pyrimidine metabolism, Ribosome, and Translation proteins. In contrast, the CN group showed significantly higher abundances of pathways associated with ABC transporters, Bacterial motility proteins, and the Two component system.

### 3.5 Intestinal untargeted metabolomic profiles

**Figure 5:**
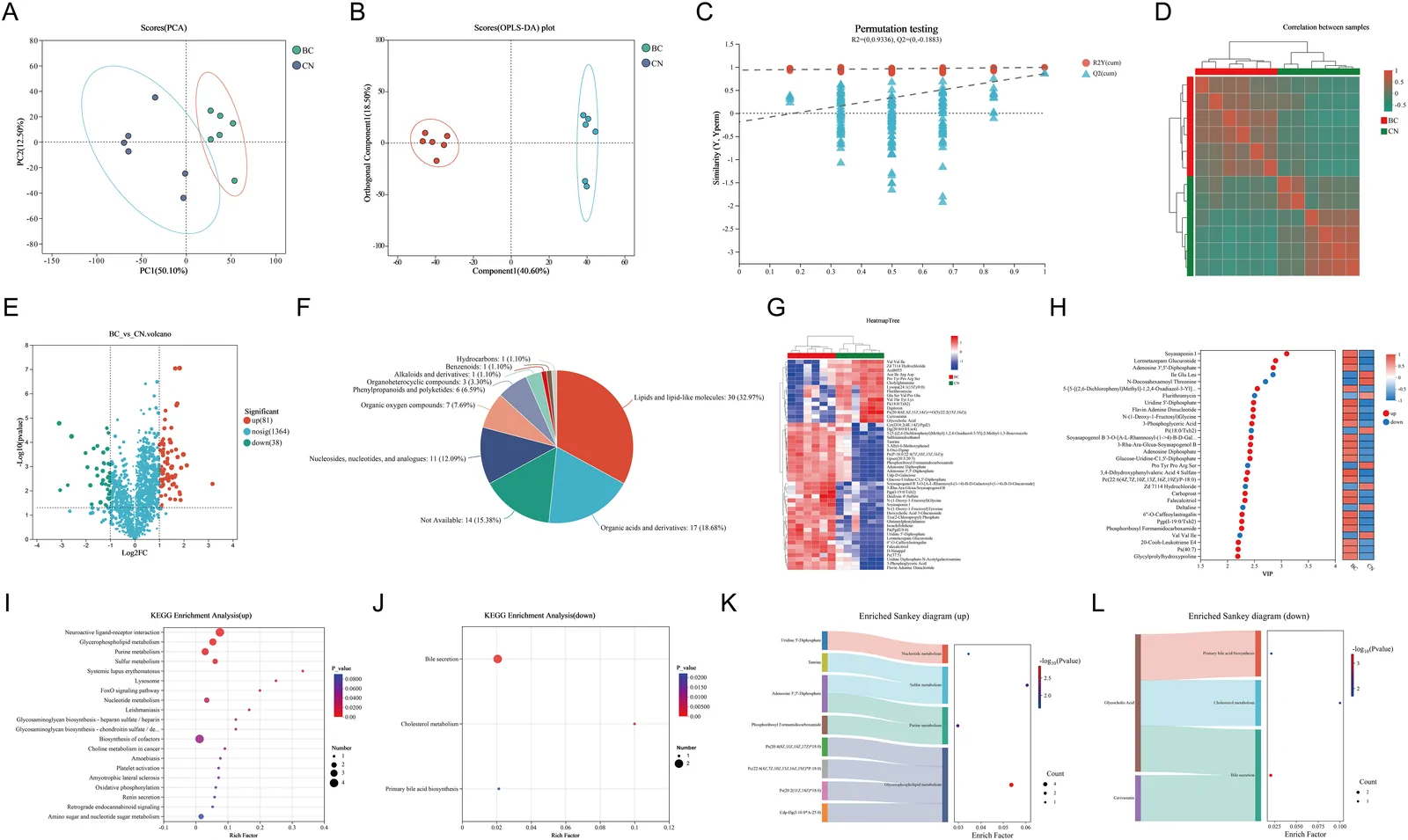
Effects of *Bacillus coagulans* on intestinal untargeted metabolomic profiles of juvenile channel catfish. **(A)** Principal component analysis (PCA) plot; **(B)** Orthogonal partial least squares-discriminant analysis (OPLS-DA) plot; **(C)** Permutation test results of OPLS-DA; **(D)** Sample-to-sample Spearman correlation heatmap; **(E)** Volcano plot of differential metabolites between groups (BC vs. CN); **(F)** HMDB-based compound classification of differential metabolites; **(G)** Clustering heatmap of differential metabolites; **(H)** Variable importance in projection (VIP) scores of differential metabolites based on the OPLS-DA model (top 30 metabolites); **(I)** KEGG pathway enrichment analysis of up-regulated differential metabolites; **(J)** KEGG pathway enrichment analysis of down-regulated differential metabolites; **(K)** Sankey diagram of up-regulated potential key metabolites and key pathways; **(L)** Sankey diagram of down-regulated potential key metabolites and key pathways.

To systematically evaluate the effects of dietary supplementation with *Bacillus coagulans* on the intestinal metabolomic profiles of juvenile channel catfish and to verify the stability of the multivariate statistical model, this study performed PCA, Spearman correlation analysis, and orthogonal partial least squares-discriminant analysis (OPLS-DA) on samples from the two groups. The PCA score plot revealed distinct clustering of samples from the CN and BC groups with no outliers, indicating clear differences in their intestinal metabolomic profiles (**Figure 5A**). The OPLS-DA analysis further revealed a clear separation between the two groups in the model, indicating significant differences in metabolomic characteristics (**Figure 5B**). The permutation test yielded R^2^ = (0, 0.9336) and Q^2^ = (0, −0.1883), indicating that the OPLS-DA model was stable and not overfitted (**Figure 5C**). In addition, the Spearman correlation heatmap revealed strong intra-group correlations and clear inter-group separation, further supporting the consistency and reproducibility of the metabolomic dataset (**Figure 5D**).

To further investigate the effects of dietary supplementation with *Bacillus coagulans* on intestinal metabolites of juvenile channel catfish, differential metabolite analysis was conducted based on VIP values derived from the OPLS-DA model, combined with multivariate statistical criteria (VIP > 1, |log_2_FC| > 1, and *P* < 0.05). A total of 119 differential metabolites were identified, including 81 significantly upregulated and 38 significantly downregulated metabolites in the BC group (**Figure 5E**). Subsequently, functional classification of the differential metabolites was conducted using the HMDB database. The results showed that Lipids and lipid-like molecules (32.97%), Organic acids and derivatives (18.68%), and Nucleosides, nucleotides, and analogues (12.09%) were the three most abundant categories among the differential metabolites (**Figure 5F**).

To more clearly illustrate the variation trends of differential metabolites between groups, hierarchical clustering analysis was performed on the 119 differential metabolites, and a clustering heatmap was constructed based on the top 50 most abundant metabolites. The results showed a clear separation between the BC and CN groups (**Figure 5G**). Finally, the top 30 differential metabolites ranked by VIP values in the OPLS-DA model were selected for focused analysis (**Figure 5H**). Among these metabolites, 22 were significantly upregulated and 8 were significantly downregulated in the BC group. Notably, several metabolites associated with intestinal health, including Soyasaponin I, Uridine 5’-Diphosphate, Flavin Adenine Dinucleotide, 3,4-Dihydroxyphenylvaleric Acid 4 Sulfate, PC(22:6(4Z,7Z,10Z,13Z,16Z,19Z)/P-18:0), Falecalcitriol, and Ps(40:7), were significantly elevated in the BC group.

To investigate the potential regulatory effects of *Bacillus coagulans* on intestinal metabolic pathways in juvenile channel catfish, KEGG pathway annotation was performed separately for the 81 upregulated and 38 downregulated differential metabolites identified in the BC group, and the top 20 significantly enriched pathways were selected for visualization. The results showed that the upregulated differential metabolites were enriched in 47 KEGG pathways, among which 13 were significantly enriched (*P* < 0.05), mainly including Glycerophospholipid metabolism, Purine metabolism, Sulfur metabolism, Lysosome, FoxO signaling pathway, and Nucleotide metabolism (**Figure 5I**). By contrast, the downregulated differential metabolites were enriched in three KEGG pathways, all of which were significantly enriched (*P* < 0.05), mainly including Primary bile acid biosynthesis, Cholesterol metabolism, and Bile secretion (**Figure 5J**).

To further identify potential key intestinal metabolites, pathways related to intestinal health, energy metabolism, and immune regulation were screened based on the KEGG enrichment results, and the relationships between these pathways and differential metabolites were visualized using Sankey diagrams (**Figures 5K, L**). Among the upregulated metabolites, Uridine 5’-Diphosphate was significantly enriched in Nucleotide metabolism; Adenosine 3’,5’-Diphosphate and Taurine were significantly enriched in Sulfur metabolism; Phosphoribosyl Formamidocarboxamide and Adenosine 3’,5’-Diphosphate were significantly enriched in Purine metabolism; and Cdp-Dg(I-16:0/A-25:0), Ps(20:2(11Z,14Z)/18:0), Pc(22:6(4Z,7Z,10Z,13Z,16Z,19Z)/P-18:0), and Ps(20:4(8Z,11Z,14Z,17Z)/18:0) were significantly enriched in Glycerophospholipid metabolism (**Figure 5K**). In contrast, among the downregulated metabolites, Glycocholic Acid was significantly enriched in Cholesterol metabolism, Primary bile acid biosynthesis, and Bile secretion, whereas Cerivastatin was additionally enriched in Bile secretion (**Figure 5L**).

## 4 Discussion

Statistical analysis of growth performance showed that, compared with the CN group, no significant differences were observed in FBW, WGR, SGR, and SR, whereas the FCR in the BC group was significantly lower than that in the CN group. These results indicated that dietary *Bacillus coagulans* improved feed efficiency in juvenile channel catfish (*Ictalurus punctatus*). Numerous studies have demonstrated that dietary supplementation with *B. coagulans* can significantly improve growth performance and reduce FCR in various aquatic animals, including Nile tilapia (15, 16), common carp (17, 38), gilthead seabream (*Sparus aurata* L.) (39), and fourfinger threadfin (*Eleutheronema tetradactylum*) (19). The absence of significant improvement in weight gain parameters in the present study, in contrast to these previous reports, may be related to species-specific differences in response to *B. coagulans*, as well as other factors such as dietary composition and rearing conditions. Further dose–response studies are needed to determine whether dietary *B. coagulans* can also improve weight gain in this species. Digestive enzyme activity is considered one of the biomarkers of intestinal health (40). In the present study, dietary supplementation with *B. coagulans* significantly increased intestinal trypsin and α-amylase activities in juvenile channel catfish. Previous studies have shown that *Bacillus* spp. can produce a variety of exogenous enzymes, including protease, β-glucanase, amylase, xylanase, phytase, lipase, and cellulase (14, 41), which may promote luminal digestion in fish. However, given that hindgut samples were thoroughly washed to remove digesta prior to analysis, the increased enzyme activities observed in this study are more likely to reflect the upregulation of host endogenous enzyme secretion, or the activity of mucosa-associated enzymes, rather than free exogenous bacterial enzymes in the lumen. For example, it has been reported that *B. coagulans* can upregulate the expression of genes related to nutrient transport and metabolism in the intestinal mucosa, thereby enhancing the ability of fish to effectively absorb essential nutrients (42). These mucosal responses may be associated with the reduction in FCR observed in fish supplemented with *B. coagulans*. The intestine is an important site of antioxidant defense in aquatic animals, and both external and internal factors can induce intestinal oxidative stress (43). Oxidative stress can lead to intestinal mucosal injury, digestive dysfunction, and inflammatory diseases (44). Therefore, intestinal antioxidant capacity plays a crucial role in maintaining intestinal function in fish (45). Antioxidant enzymes, such as CAT, GST, and T-SOD, constitute the first line of defense against oxidative stress (46), whereas the levels of MDA and O_2_^−^ reflect the organism’s ability to scavenge free radicals (47). In the present study, dietary supplementation with *B. coagulans* significantly enhanced intestinal GST activity while reducing MDA and O_2_^−^ contents, indicating that it effectively improved intestinal antioxidant capacity. This result is consistent with previous reports (16, 48, 49). This effect may be attributed to the ability of *B. coagulans*, as an antigenic stimulus, to promote the secretion of antioxidant defense enzymes, thereby alleviating oxidative stress (50, 51) and contributing to the repair of tissue and organ damage (39). These findings further suggested that *B. coagulans* played an important role in enhancing host antioxidant capacity and reducing oxidative damage. This may represent one of the potential mechanisms by which *B. coagulans* promotes intestinal functional stability in channel catfish.

The integrity of intestinal mucosal barrier function is crucial for the health of aquatic animals. Tight junction proteins, such as *zo-2*, *claudins*, and *jams*, are key components of the intestinal mechanical barrier. They are responsible for maintaining the tightness of intercellular junctions between intestinal epithelial cells, regulating mucosal barrier permeability, and preventing the invasion of pathogens and toxins (52). In the present study, dietary supplementation with *B. coagulans* significantly upregulated the expression of tight junction protein genes, including *zo-2*, *claudin-18*, *claudin-g*, *jam-2b*, and *jam-3b*, in the intestine of juvenile channel catfish. Previous studies have shown that *B. coagulans* can significantly enhance tight junction gene expression and strengthen the structure and function of the intestinal epithelial barrier through interactions with host cell junctional complexes (53, 54). Similar findings have also been reported for other probiotics used in feed; for example, lactic acid bacteria and *Bacillus subtilis* have both been shown to enhance intestinal tight junction protein expression (55). The inflammatory response is a key immune process in host defense against pathogen infection, and the dynamic balance between pro-inflammatory and anti-inflammatory factors determines intestinal immune homeostasis (45, 56). In the present study, dietary supplementation with *B. coagulans* significantly downregulated the expression levels of pro-inflammatory cytokine genes (*il-1β*, *tnf-α*, *il-6*, and *ifn-γ1*) and significantly upregulated the expression level of the anti-inflammatory cytokine gene *tgf-β* in the intestine of juvenile channel catfish, suggesting that it can effectively suppress intestinal inflammatory responses. This result is consistent with reports in other aquatic animals. For example, *B. coagulans* has been shown to alleviate cadmium-induced inflammation in common carp (20) and to reduce inflammatory factor levels in Nile tilapia (16) and common carp (38). Regarding the chemical barrier, dietary supplementation with *B. coagulans* significantly upregulated the expression levels of *mucin-4*, *lysozyme-c*, *lysozyme-g*, *NK-lysin type 1*, and *β-defensin* in the intestine of juvenile channel catfish. Mucins are primarily secreted by intestinal goblet cells and form a protective layer on the intestinal mucosal surface, thereby preventing pathogen adhesion and invasion and maintaining intestinal microecological homeostasis (57). Lysozyme directly kills pathogens by degrading the peptidoglycan structure of bacterial cell walls (58). In addition, antimicrobial peptides, such as *NK-lysin type 1* and *β-defensin*, can resist infection by a variety of pathogenic microorganisms, thereby promoting health and enhancing disease prevention (59). Collectively, dietary supplementation with *B. coagulans* may contribute to the enhancement of the mechanical, immune, and chemical barrier functions of the intestine in juvenile channel catfish by upregulating the expression of tight junction protein genes, suppressing the expression of pro-inflammatory cytokine genes while increasing the expression of anti-inflammatory cytokine genes, and increasing the expression of chemical barrier-related genes. However, it should be noted that a complex regulatory relationship exists between gene expression levels and protein abundance. Up-regulated gene expression levels cannot be directly equated to an increase in the corresponding protein quantity. Future studies incorporating other methods (for example, proteomic and immunohistochemical validation) would be necessary to fully elucidate the functional implications of these gene expression changes.

The present study found that dietary supplementation with *B. coagulans* significantly affected the diversity and composition of the intestinal microbiota in juvenile channel catfish. Numerous studies have shown that the diversity and composition of the intestinal microbiota directly influence host physiology, metabolism, and immune function (13, 60, 61). In the present study, among the dominant members of the intestinal microbiota (the top ten bacterial phyla and genera ranked by relative abundance), dietary supplementation with *B. coagulans* tended to increase the relative abundances of Fusobacteriota, Firmicutes, and *Cetobacterium*. Previous studies have shown that Fusobacteriota can promote host lipid and glucose metabolism through the production of butyrate, thereby providing energy for intestinal cells and modulating intestinal immunity (62). Similarly, it has been reported that Firmicutes, particularly Clostridium species, can produce short-chain fatty acids such as butyrate through the fermentation of proteins and carbohydrates, thereby improving intestinal epithelial cell function and barrier integrity (62, 63). In addition, it has been documented that *Cetobacterium* strains can not only produce butyrate but also synthesize vitamin B_12_ (64). Furthermore, LEfSe analysis revealed that the *B. coagulans*-supplemented group was specifically enriched in taxa such as Bacilli, *Turicibacter*, Erysipelotrichaceae, and Firmicutes. Among these taxa, Bacilli have been shown to produce short-chain fatty acids and other antimicrobial metabolites, thereby playing a positive role in maintaining intestinal homeostasis (20). In mammals, *Turicibacter* has been reported to reduce serum cholesterol and triglyceride levels and to regulate ROS-mediated apoptosis (65). Erysipelotrichaceae has been associated with inflammatory diseases and can produce broad-spectrum antimicrobial substances to inhibit the growth of other pathogenic bacteria (66). LEfSe analysis also revealed that the control group was significantly enriched in potentially harmful bacteria, such as Proteobacteria and Planctomycetota. Proteobacteria are generally closely associated with intestinal inflammation, microbial dysbiosis, and intestinal diseases (67, 68), whereas Planctomycetota has been reported to increase in abundance in the intestine of diseased grass carp (69). Therefore, these shifts in the relative abundances of these bacterial taxa indicated that dietary supplementation with *B. coagulans* can effectively improve host intestinal health. Functional prediction analysis of the intestinal microbiota further showed that, following *B. coagulans* supplementation, multiple key pathways related to genetic information processing and basic metabolism were predicted to be significantly upregulated, including DNA repair and recombination proteins, Purine metabolism, Pyrimidine metabolism, Amino sugar and nucleotide sugar metabolism, Energy metabolism, and Citrate cycle (TCA cycle). Predicted activation of genetic information processing pathways, such as DNA repair and recombination proteins, suggests an increased microbial metabolic potential that could theoretically support the repair of oxidative stress-induced DNA damage (70). In addition, the predicted enhancement of energy and nutrient metabolism-related pathways, such as Amino sugar and nucleotide sugar metabolism, Energy metabolism, and Citrate cycle, suggests a higher microbial capacity for substrate utilization, which may indirectly benefit host nutrient metabolism and stress resistance (71). Collectively, *B. coagulans* showed the potential to optimize the intestinal microbiota and its predicted functional potential in juvenile channel catfish, which may contribute to improved intestinal health.

The functions of intestinal bacteria are closely associated with their metabolites, and metabolomic analysis contributes to a deeper understanding of the mechanisms of action of *B. coagulans*. In the present study, metabolomic analysis revealed that the upregulated differential metabolites in the BC group were primarily enriched in pathways such as Neuroactive ligand-receptor interaction, Glycerophospholipid metabolism, Purine metabolism, and Sulfur metabolism. Various phospholipid components detected in the Glycerophospholipid metabolism pathway, such as Cdp-Dg(I 16:0/A 25:0) and Ps(20:2/18:0), are important structural lipids of cell membranes and the mucosal barrier (72). Increased phospholipid levels may potentially contribute to the fluidity and stability of epithelial tight junctions, thereby supporting the integrity of the intestinal mechanical barrier (73, 74). Moreover, phospholipid metabolism also provides secondary signaling molecules for intestinal epithelial cells, such as diacylglycerol (DAG) and lysophosphatidic acid (LPA), which participate in immune signal transduction and mucosal repair (75). This observation is consistent with previous reports showing that *B. coagulans* can upregulate the expression of tight junction proteins such as *occludin* and *claudin-1* in multiple hosts (53, 54). Purine metabolism showed an upward trend, and the increase in purine substrates might provide sufficient nucleotide precursors for rapidly renewing intestinal epithelial cells, thereby potentially promoting DNA/RNA synthesis and mucosal regeneration (76). In the Sulfur metabolism pathway, taurine was significantly upregulated. Taurine not only serves as a bile acid conjugate but also functions as an osmotic regulator and antioxidant (77). In addition, elevated taurine levels are usually closely associated with intestinal immunomodulation, anti-inflammatory effects, and enhanced expression of tight junction proteins (78, 79). The downregulated differential metabolites in the BC group were mainly enriched in pathways such as Bile secretion, Cholesterol metabolism, and Primary bile acid biosynthesis. As a conjugated primary bile acid, reduced synthesis of glycocholic acid might affect intestinal health through the following two hypothetical mechanisms. On the one hand, a reduction in glycocholic acid might attenuate intestinal epithelial cell injury by inhibiting the excessive conversion of cholesterol into bile acids and reducing endoplasmic reticulum stress (80). On the other hand, a decrease in primary bile acid levels might reduce the conversion of glycocholic acid by the intestinal microbiota into cytotoxic secondary bile acids, such as deoxycholic acid, thereby potentially alleviating oxidative damage to intestinal epithelial cells (81). In addition, the downregulation of the statin drug cerivastatin is an intriguing finding that might suggest an indirect inhibitory effect of *B. coagulans* on HMG-CoA reductase, the rate-limiting enzyme in cholesterol synthesis, although this interpretation remains highly speculative (82). If confirmed, this effect could be achieved through competitive consumption of precursors in the mevalonate pathway, thereby reducing the risk of cholesterol accumulation in intestinal cells, enhancing cell membrane fluidity, and helping maintain nutrient absorption and signal transduction functions (83). Collectively, these metabolomic changes suggested that *B. coagulans* had the potential to modulate multiple metabolic pathways in a manner that could favor intestinal homeostasis and mucosal barrier function. These findings provided preliminary metabolomic evidence and a theoretical basis for the healthy growth of channel catfish and highlighted specific metabolic targets for future investigation into the mechanisms by which *B. coagulans* improves intestinal health. Nevertheless, it should be emphasized that these metabolomic findings are correlative in nature, and the proposed metabolic mechanisms remain to be validated through targeted interventional studies.

## 5 Conclusion

Although dietary supplementation with *Bacillus coagulans* did not significantly improve the growth performance of juvenile channel catfish, it significantly reduced feed conversion ratio, enhanced intestinal digestive and antioxidant capacity, strengthened intestinal mucosal barrier function by improving expression levels of genes related to the mechanical, chemical, and immune barriers, and improved the intestinal microbiota and intestinal metabolism. These findings provide systematic experimental evidence and support the application of *B. coagulans* for improving intestinal health and promoting growth in channel catfish.

## Data Availability

The raw data supporting the conclusions of this article will be made available by the corresponding author.
