# Supplementary material for: Effects of Bacillus coagulans on growth performance and intestinal mucosal barrier functions of juvenile channel catfish (Ictalurus punctatus)
Source: Front Immunol. 2026 Sep 3;17:1909579. doi: 10.3389/fimmu.2026.1909579 (PMC13581747; doi:10.3389/fimmu.2026.1909579)
Supplement: Supplementary file 1 [file DataSheet1.docx]

Supplementary Material

# 1 Supplementary Figures

**Supplementary Figure 1.** Rarefaction curve based on the Shannon index of intestinal microbiota in juvenile channel catfish.

**Supplementary Figure 2.** Rarefaction curve based on the Simpson index of intestinal microbiota in juvenile channel catfish.

**Supplementary Figure 3.** Effects of *Bacillus coagulans* on Good's coverage index of intestinal microbiota in juvenile channel catfish.
